# 6-(3,5-Dimethyl-1*H*-pyrazol-1-yl)-1,2,4,5-tetra­zin-3(2*H*)-one

**DOI:** 10.1107/S1600536813027360

**Published:** 2013-10-12

**Authors:** Kyrill Yu. Suponitsky, Victor M. Chernyshev, Anna G. Mazharova, Nadezhda V. Palysaeva, Aleksei B. Sheremetev

**Affiliations:** a A. N. Nesmeyanov Institute of Organoelement Compounds, Russian Academy of Sciences, 28 Vavilova St, 119991 Moscow, Russian Federation; b South-Russia State Technical University, 346428 Novocherkassk, Russian Federation; c N. D. Zelinsky Institute of Organic Chemistry, Russian Academy of Sciences, 47 Leninsky Prosp, 119991 Moscow, Russian Federation

## Abstract

The title compound, C_7_H_8_N_6_O, represents the keto form and adopts a nearly planar structure (r.m.s. deviation of the non-H atoms = 0.072 Å). In the crystal, mol­ecules form spiral chains along the *c* axis by N—H⋯N hydrogen bonds. The chains are linked to each other by weak C—H⋯O hydrogen bonds, forming a three-dimensional framework.

## Related literature

For review on nucleophilic displacement at the 1,2,4,5-tetra­zine ring, see: Clavier & Audebert (2010 ▶); Tolshchina *et al.* (2013 ▶). For the synthesis of 3-hy­droxy-1,2,4,5-tetra­zines, see: Ishmetova *et al.* (2009 ▶); Sheremetev *et al.* (2012*a* ▶,*b* ▶). For the structure of 3-hy­droxy-1,2,4,5-tetra­zine, see: Yeh *et al.* (1994 ▶). For a review on oxo-hy­droxy tautomerism of various azines, see: Stanovnik *et al.* (2006 ▶). For standard bond lengths, see: Allen *et al.* (1987 ▶).
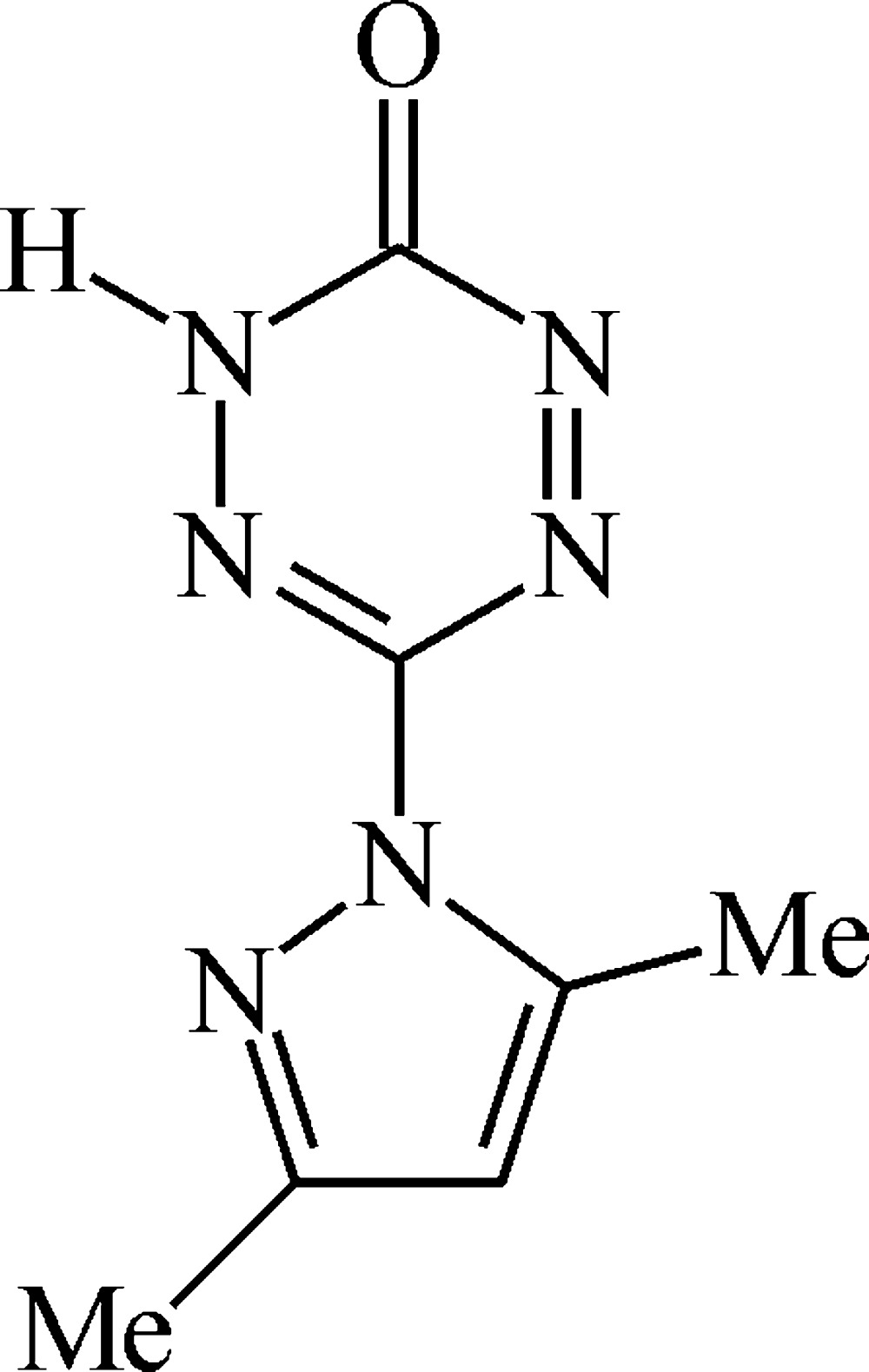


## Experimental

### 

#### Crystal data

C_7_H_8_N_6_O
*M* *_r_* = 192.19
Orthorhombic, 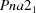

*a* = 12.1431 (11) Å
*b* = 12.6551 (12) Å
*c* = 5.3907 (5) Å
*V* = 828.40 (13) Å^3^
*Z* = 4
Mo *K*α radiation
μ = 0.11 mm^−1^
*T* = 120 K
0.28 × 0.22 × 0.20 mm

#### Data collection

Bruker APEXII CCD diffractometer
14157 measured reflections
1353 independent reflections
1228 reflections with *I* > 2σ(*I*)
*R*_int_ = 0.050

#### Refinement

*R*[*F* ^2^ > 2σ(*F* ^2^)] = 0.037
*wR*(*F* ^2^) = 0.094
*S* = 1.05
1353 reflections
133 parameters
1 restraint
H atoms treated by a mixture of independent and constrained refinement
Δρ_max_ = 0.40 e Å^−3^
Δρ_min_ = −0.28 e Å^−3^

### 

Data collection: *APEX* (Bruker, 2009 ▶); cell refinement: *SAINT* (Bruker, 2009 ▶); data reduction: *SAINT*; program(s) used to solve structure: *SHELXTL* (Sheldrick, 2008 ▶); program(s) used to refine structure: *SHELXTL*; molecular graphics: *SHELXTL*; software used to prepare material for publication: *SHELXTL*, *PLATON* (Spek, 2009 ▶) and *publCIF* (Westrip, 2010 ▶).

## Supplementary Material

Crystal structure: contains datablock(s) I, New_Global_Publ_Block. DOI: 10.1107/S1600536813027360/kq2010sup1.cif

Structure factors: contains datablock(s) I. DOI: 10.1107/S1600536813027360/kq2010Isup2.hkl

Supplementary material file. DOI: 10.1107/S1600536813027360/kq2010Isup3.cml

Additional supplementary materials:  crystallographic information; 3D view; checkCIF report

## Figures and Tables

**Table 1:** Hydrogen-bond geometry (Å, °)

*D*—H⋯*A*	*D*—H	H⋯*A*	*D*⋯*A*	*D*—H⋯*A*
N4—H4⋯N1^i^	0.89 (3)	2.00 (3)	2.863 (2)	161 (3)
C2—H2*A*⋯O1^ii^	0.95	2.40	3.193 (3)	141

Symmetry codes: (i) 
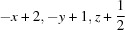
; (ii) 
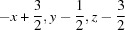
.

**Fig. 1.**
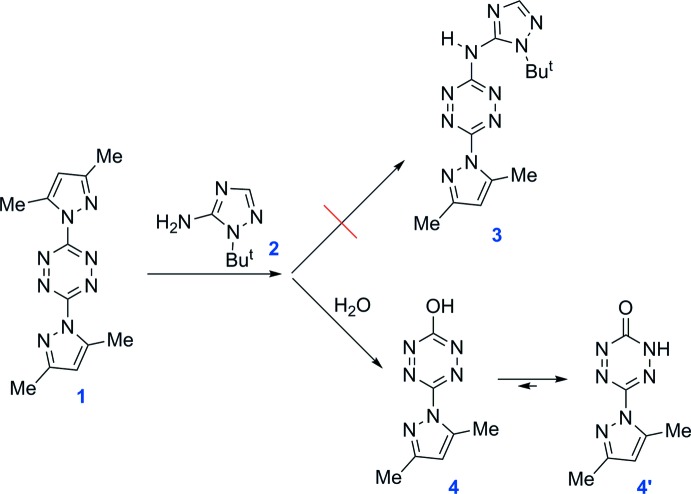
Synthesis of the title compound.

**Fig. 2.**
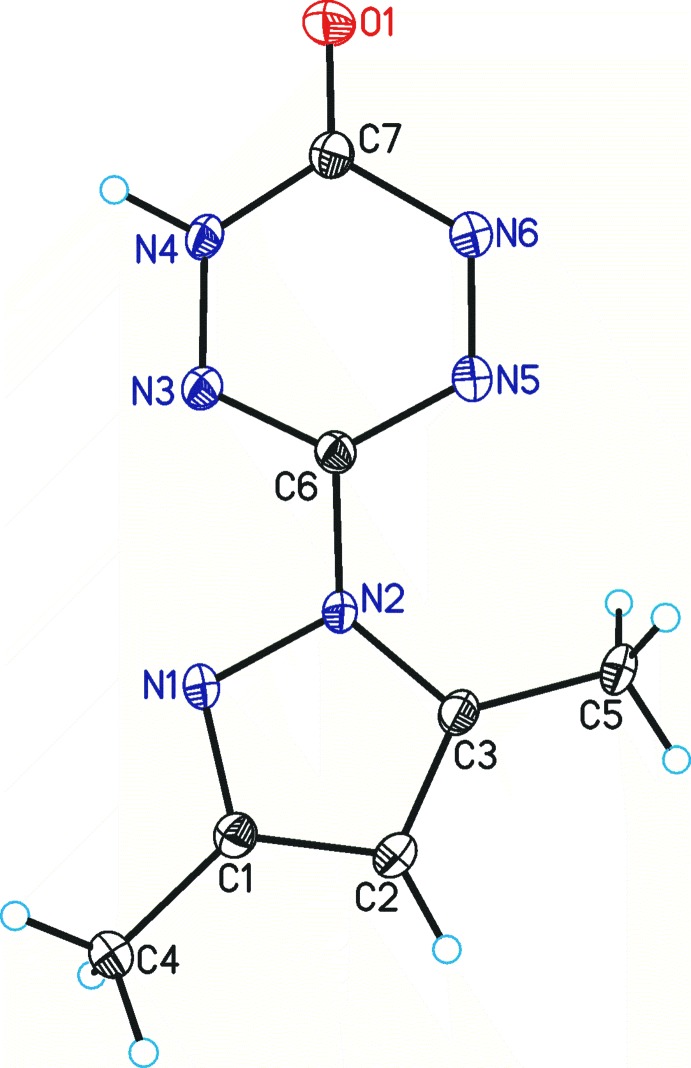
General view of the structure of the title compound with the atomic numbering scheme. Displacement ellipsoids are drawn at the 50% probability level. H atoms are presented as small spheres of arbitrary radius.

**Fig. 3.**
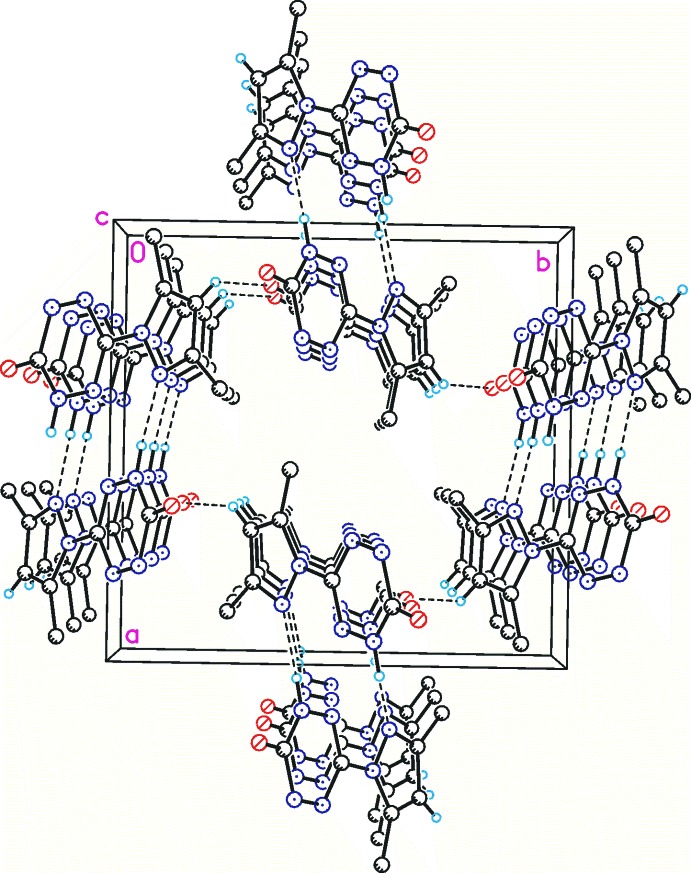
Crystal packing fragment of the title compound. Hydrogen atoms of the methyl groups are omitted for clarity. Dashed lines indicate the intermolecular N—H···N and C—H···O hydrogen bonds.

## supplementary crystallographic information

### 1. Comment

Over the last decade, broad studies of nucleophilic displacemet at 1,2,4,5-tetrazine ring have demonstrated that the highly electron-withdrawing aromatic ring exhibit a rich diversity of chemical behavior, and today 3,6-bis(3,5-dimethylpyrazol-1-yl)-1,2,4,5-tetrazine 1 has become common precursor to various tetrazine derivatives (Clavier & Audebert, 2010; Tolshchina *et al.*, 2013). The displacement of the dimethylpyrazolyl moiety in compound 1 by a wider range of *N*-nucleophiles has been well documented (Sheremetev *et al.*, 2012*a*, 2012*b*), while reactions of the tetrazine 1 with *O*-nucleophiles have been studied to a lesser extent (Yeh *et al.*, 1994; Ishmetova *et al.*, 2009). Here, we wish to report the unexpected discovery of an unusual dimethylpyrazolyl moiety displacement of compound 1 when it is treated with *N*-nucleophile, such as 3-amino-1-*tret-*butyl-1,2,4-triazole 2.

Upon a refluxing of compound 1 with amine 2 and K_2_CO_3_ in acetonitrile and following treatment with water, an step required to workup of the reaction mixture, it was found that instead of introduction of *N*-nucleophile (with a formation of compound 3) the reaction gave the hydroxy derivative 4' (Figure 1).

According to X-ray data, the title compound adopts nearly planar structure (r.m.s. deviation of the non-hydrogen atoms is 0.072 Å) (Figure 2). In general, it can exist in two tautomeric forms (Stanovnik *et al.*, 2006) as shown in Figure 1. The hydrogen atom H4 was localized and refined at the N4 nitrogen atom that means that the title compound exists in keto-form, and the equilibrium favors the tautomer 4'. This is also supproted by the bond length distribution in the tetrazin-3-one: the C7—O1 (1.215 (3) Å) bond corresponds to normal carbonyl bond (standard X-ray C═O value is in the range of 1.192–1.235 Å (Allen *et al.* 1987), while the N3—C6 (1.300 (2) Å) and N5—N6 (1.286 (2) Å) bonds are significantly shorter than the C7—N4 (1.368 (3) Å), N3—N4 (1.384 (2) Å), C6—N5 (1.377 (2) Å) and N6—C7 (1.427 (2) Å) bonds.

The crystal structure of 4' is stabilized by intermolecular N—H···N and C—H···O hydrogen bonds (Table 1). By means of the N4—H4···N1^i^ hydrogen bond, molecules are connected into spiral chains along the *c* axis (Figure 3). Those chains are linked into 3D-framework (Figure 3) by the C2—H2A···O1^ii^ contacts. Symmetry codes: (i) –*x*+2, –*y*+1, *z*+1/2; (ii) –*x*+3/2, *y*–1/2, *z*–3/2.

### 2. Experimental

The X-ray quality crystals of the title compound were grown by slow evaporation of acetonitrile solution.

All the reagents were of analytical grade, purchased from commercial sources, and used as received. Infrared spectra were determined in KBr pellets on a Perkin-Elmer Model 577 spectrometer. Mass-spectra were recorded on a Varian MAT-311 A instrument. The ^1^H, ^13^C and ^15^N NMR spectra (external standard: CH_3_NO_2_) were recorded at 300.13, 75.47 and 50.7 MHz, respectively. The chemical shift values (*δ*, p.p.m.) are expressed relative to the chemical shift of the solvent-*d* or to external standard without correction nitromethane (^14^N and ^15^N). Melting points were determined on Gallenkamp melting point apparatus and are uncorrected.

6-(3,5-Dimethyl-1*H*-pyrazol-1-yl)-1,2,4,5-tetrazin-3(2*H*)-one (4'). A mixture of 3,6-bis(3,5-dimethylpyrazol-1-yl)-1,2,4,5-tetrazine 1 (0.27 g, 1 mmol), 3-amino-1-*tret*butyl-1,2,4-triazole 2 (0.14 g, 1 mmol), and K_2_CO_3_ (0.14 g, 1 mmol) was refluxed in dry acetonitrile (10 mL) for 3 d. The reaction mixture was then cooled and a violet solid formed was collected by filtration. The solid was dissolved in water (10 mL), acidifed with 3% HCl to pH 1 and cooled to 278 K. A solid was collected by filtration, washed with 1% HCl and hexane and crystallized from MeCN/H_2_O, yield 0.15 g (82%), mp 483–484 K (dec.); IR (KBr, cm^-1^): ν = 3135–2823, 1744, 1706, 1693, 1553, 1506, 1435, 1405, 1373, 1263, 1161, 1138, 1105, 1063, 1025, 993, 975, 836, 808. ^1^H MNR (DMSO-*d*_3_, 293 K): *δ* = 2.20 and 2.38 (3H+3H, C1—CH_3_ and C3—CH_3_), 6.17 (c, 1H, CH), 8.51 (br, 1H, OH); ^13^C MNR (DMSO-*d*_3_, 293 K): *δ =* 13.3, 13.2, 108.9, 141.9, 147.7, 150.7, 152.0. ^15^N MNR (DMSO-*d*_3_, 293 K): *δ* = –1.87, –35.0, –77.6, –173.3. Anal. Calcd. for C_7_H_8_N_6_O (192.17): C, 43.75; H, 4.20; N, 43.73. Found: C, 43.81; H, 4.24; N, 43.61.

### 3. Refinement

The hydrogen atom of the NH group was found in difference Fourier synthesis and refined in isotropic approximation. The H(C) atomic positions were calculated and refined in isotropic approximation in riding model with the *U*_iso_(H) parameters equal to 1.5 *U*_eq_(Ci), 1.2 *U*_eq_(Cj), where *U*_eq_(Ci) and *U*_eq_(Cj) are the equivalent thermal parameters of the methyl carbon atoms and all the other carbon atoms, respectively, to which corresponding H atoms are bonded.

### Crystal data

**Table:**

C_7_H_8_N_6_O	*D*_x_ = 1.541 Mg m^−^^3^
*M**_r_* = 192.19	Melting point = 474–473 K
Orthorhombic, *P**n**a*2_1_	Mo *K*α radiation, λ = 0.71073 Å
Hall symbol: P 2c -2n	Cell parameters from 4489 reflections
*a* = 12.1431 (11) Å	θ = 2.3–30.2°
*b* = 12.6551 (12) Å	µ = 0.11 mm^−^^1^
*c* = 5.3907 (5) Å	*T* = 120 K
*V* = 828.40 (13) Å^3^	Prizm, red
*Z* = 4	0.28 × 0.22 × 0.20 mm
*F*(000) = 400	

### Data collection

**Table:**

Bruker APEXII CCD diffractometer	1228 reflections with *I* > 2σ(*I*)
Radiation source: sealed tube	*R*_int_ = 0.050
Graphite monochromator	θ_max_ = 30.2°, θ_min_ = 2.3°
φ and ω scans	*h* = −17→17
14157 measured reflections	*k* = −17→17
1353 independent reflections	*l* = −7→7

### Refinement

**Table:**

Refinement on *F*^2^	Primary atom site location: structure-invariant direct methods
Least-squares matrix: full	Secondary atom site location: difference Fourier map
*R*[*F*^2^ > 2σ(*F*^2^)] = 0.037	Hydrogen site location: mixed
*wR*(*F*^2^) = 0.094	H atoms treated by a mixture of independent and constrained refinement
*S* = 1.05	*w* = 1/[σ^2^(*F*_o_^2^) + (0.0529*P*)^2^ + 0.2463*P*] where *P* = (*F*_o_^2^ + 2*F*_c_^2^)/3
1353 reflections	(Δ/σ)_max_ < 0.001
133 parameters	Δρ_max_ = 0.40 e Å^−^^3^
1 restraint	Δρ_min_ = −0.28 e Å^−^^3^

### Special details

**Table:**

Geometry. All e.s.d.'s (except the e.s.d. in the dihedral angle between two l.s. planes) are estimated using the full covariance matrix. The cell e.s.d.'s are taken into account individually in the estimation of e.s.d.'s in distances, angles and torsion angles; correlations between e.s.d.'s in cell parameters are only used when they are defined by crystal symmetry. An approximate (isotropic) treatment of cell e.s.d.'s is used for estimating e.s.d.'s involving l.s. planes.
Refinement. Refinement of *F*^2^ against ALL reflections. The weighted *R*-factor *wR* and goodness of fit *S* are based on *F*^2^, conventional *R*-factors *R* are based on *F*, with *F* set to zero for negative *F*^2^. The threshold expression of *F*^2^ > σ(*F*^2^) is used only for calculating *R*-factors(gt) *etc*. and is not relevant to the choice of reflections for refinement. *R*-factors based on *F*^2^ are statistically about twice as large as those based on *F*, and *R*- factors based on ALL data will be even larger.

### Fractional atomic coordinates and isotropic or equivalent isotropic displacement parameters (Å^2^)

**Table:**

	*x*	*y*	*z*	*U*_iso_*/*U*_eq_	
O1	0.86033 (12)	0.65658 (12)	1.3631 (3)	0.0238 (3)	
N1	0.85424 (13)	0.38253 (13)	0.4472 (3)	0.0155 (3)	
N2	0.76721 (12)	0.41809 (12)	0.5895 (3)	0.0144 (3)	
N3	0.89653 (13)	0.50888 (12)	0.8195 (3)	0.0158 (3)	
N4	0.91664 (13)	0.57202 (12)	1.0123 (3)	0.0155 (3)	
H4	0.989 (2)	0.583 (2)	1.029 (7)	0.034 (7)*	
N5	0.70626 (13)	0.52560 (13)	0.9220 (3)	0.0188 (4)	
N6	0.72745 (13)	0.58417 (14)	1.1110 (4)	0.0202 (4)	
C1	0.80934 (15)	0.31818 (14)	0.2799 (4)	0.0148 (3)	
C2	0.69351 (15)	0.31263 (15)	0.3124 (4)	0.0173 (4)	
H2A	0.6432	0.2729	0.2149	0.021*	
C3	0.66818 (15)	0.37567 (14)	0.5118 (4)	0.0156 (4)	
C4	0.87981 (15)	0.26232 (15)	0.0946 (4)	0.0186 (4)	
H4A	0.9493	0.3008	0.0741	0.028*	
H4B	0.8413	0.2591	−0.0650	0.028*	
H4C	0.8951	0.1905	0.1529	0.028*	
C5	0.55864 (15)	0.39560 (17)	0.6288 (4)	0.0227 (4)	
H5A	0.5624	0.3785	0.8059	0.034*	
H5B	0.5028	0.3512	0.5489	0.034*	
H5C	0.5389	0.4702	0.6085	0.034*	
C6	0.79310 (14)	0.48639 (14)	0.7861 (4)	0.0139 (3)	
C7	0.83879 (16)	0.60685 (15)	1.1765 (4)	0.0168 (4)	

### Atomic displacement parameters (Å^2^)

**Table:**

	*U*^11^	*U*^22^	*U*^33^	*U*^12^	*U*^13^	*U*^23^
O1	0.0219 (7)	0.0287 (7)	0.0207 (7)	0.0014 (6)	−0.0019 (6)	−0.0093 (6)
N1	0.0105 (6)	0.0199 (7)	0.0162 (7)	−0.0001 (6)	0.0027 (6)	−0.0025 (6)
N2	0.0093 (6)	0.0197 (7)	0.0144 (7)	−0.0004 (5)	0.0012 (6)	−0.0029 (6)
N3	0.0123 (7)	0.0206 (7)	0.0145 (8)	0.0001 (6)	−0.0012 (6)	−0.0022 (6)
N4	0.0100 (6)	0.0205 (7)	0.0161 (7)	0.0001 (6)	−0.0020 (6)	−0.0016 (6)
N5	0.0125 (7)	0.0223 (8)	0.0216 (8)	0.0002 (6)	0.0015 (7)	−0.0056 (7)
N6	0.0145 (7)	0.0240 (8)	0.0221 (9)	−0.0001 (6)	0.0015 (7)	−0.0072 (7)
C1	0.0141 (8)	0.0154 (7)	0.0147 (8)	−0.0008 (6)	0.0000 (7)	0.0013 (7)
C2	0.0133 (8)	0.0194 (8)	0.0192 (9)	−0.0028 (6)	−0.0020 (7)	−0.0022 (8)
C3	0.0118 (8)	0.0187 (8)	0.0164 (8)	−0.0010 (6)	−0.0013 (7)	−0.0003 (7)
C4	0.0160 (8)	0.0227 (9)	0.0171 (8)	0.0004 (7)	0.0022 (8)	−0.0029 (7)
C5	0.0092 (8)	0.0326 (10)	0.0263 (11)	−0.0013 (7)	−0.0008 (8)	−0.0071 (9)
C6	0.0131 (8)	0.0162 (8)	0.0125 (8)	0.0001 (6)	0.0008 (7)	−0.0003 (7)
C7	0.0149 (8)	0.0180 (8)	0.0176 (9)	0.0011 (6)	−0.0013 (7)	−0.0004 (7)

### Geometric parameters (Å, º)

**Table:**

O1—C7	1.215 (3)	C1—C2	1.419 (2)
N1—C1	1.332 (3)	C1—C4	1.494 (3)
N1—N2	1.381 (2)	C2—C3	1.373 (3)
N2—C3	1.382 (2)	C2—H2A	0.9500
N2—C6	1.403 (2)	C3—C5	1.494 (3)
N3—C6	1.300 (2)	C4—H4A	0.9800
N3—N4	1.334 (2)	C4—H4B	0.9800
N4—C7	1.368 (3)	C4—H4C	0.9800
N4—H4	0.89 (3)	C5—H5A	0.9800
N5—N6	1.286 (2)	C5—H5B	0.9800
N5—C6	1.377 (2)	C5—H5C	0.9800
N6—C7	1.427 (2)		
			
C1—N1—N2	105.18 (15)	C1—C4—H4A	109.5
N1—N2—C3	111.78 (16)	C1—C4—H4B	109.5
N1—N2—C6	116.66 (14)	H4A—C4—H4B	109.5
C3—N2—C6	131.54 (16)	C1—C4—H4C	109.5
C6—N3—N4	114.58 (15)	H4A—C4—H4C	109.5
N3—N4—C7	124.80 (16)	H4B—C4—H4C	109.5
N3—N4—H4	111 (2)	C3—C5—H5A	109.5
C7—N4—H4	124 (2)	C3—C5—H5B	109.5
N6—N5—C6	118.45 (15)	H5A—C5—H5B	109.5
N5—N6—C7	120.11 (17)	C3—C5—H5C	109.5
N1—C1—C2	110.64 (17)	H5A—C5—H5C	109.5
N1—C1—C4	120.53 (17)	H5B—C5—H5C	109.5
C2—C1—C4	128.82 (17)	N3—C6—N5	125.96 (16)
C3—C2—C1	106.84 (17)	N3—C6—N2	117.11 (15)
C3—C2—H2A	126.6	N5—C6—N2	116.91 (15)
C1—C2—H2A	126.6	O1—C7—N4	123.63 (18)
C2—C3—N2	105.54 (16)	O1—C7—N6	120.87 (18)
C2—C3—C5	128.92 (17)	N4—C7—N6	115.47 (18)
N2—C3—C5	125.54 (18)		
			
C1—N1—N2—C3	0.3 (2)	C6—N2—C3—C5	0.5 (3)
C1—N1—N2—C6	178.57 (15)	N4—N3—C6—N5	3.3 (3)
C6—N3—N4—C7	4.1 (3)	N4—N3—C6—N2	−178.47 (16)
C6—N5—N6—C7	−0.3 (3)	N6—N5—C6—N3	−5.2 (3)
N2—N1—C1—C2	0.4 (2)	N6—N5—C6—N2	176.59 (18)
N2—N1—C1—C4	−178.72 (16)	N1—N2—C6—N3	−1.0 (2)
N1—C1—C2—C3	−1.0 (2)	C3—N2—C6—N3	176.88 (19)
C4—C1—C2—C3	178.11 (19)	N1—N2—C6—N5	177.36 (16)
C1—C2—C3—N2	1.0 (2)	C3—N2—C6—N5	−4.7 (3)
C1—C2—C3—C5	−178.20 (19)	N3—N4—C7—O1	173.22 (18)
N1—N2—C3—C2	−0.8 (2)	N3—N4—C7—N6	−8.9 (3)
C6—N2—C3—C2	−178.82 (18)	N5—N6—C7—O1	−175.41 (19)
N1—N2—C3—C5	178.44 (18)	N5—N6—C7—N4	6.6 (3)

### Hydrogen-bond geometry (Å, º)

**Table:**

*D*—H···*A*	*D*—H	H···*A*	*D*···*A*	*D*—H···*A*
N4—H4···N1^i^	0.89 (3)	2.00 (3)	2.863 (2)	161 (3)
C2—H2*A*···O1^ii^	0.95	2.40	3.193 (3)	141

Symmetry codes: (i) −*x*+2, −*y*+1, *z*+1/2; (ii) −*x*+3/2, *y*−1/2, *z*−3/2.
